# Trends and determinants of early initiation of breastfeeding in Indonesia: A multivariate decomposition analysis

**DOI:** 10.1371/journal.pone.0294900

**Published:** 2023-11-28

**Authors:** Siti Nurokhmah, Lucinda Middleton, Judhiastuty Februhartanty, Aryono Hendarto

**Affiliations:** 1 Department of Nutrition, Faculty of Medicine, Universitas Indonesia Indonesia–Dr. Cipto Mangunkusumo National Central Hospital, Jakarta, Indonesia; 2 Department of Nutrition Science, Faculty of Health Science, Universitas Muhammadiyah Surakarta, Surakarta, Indonesia; 3 Research Institute for the Environment and Livelihoods, Charles Darwin University, Ellengowan Drive, Australia; 4 South-East Asian Ministers of Education Organization Regional Centre for Food and Nutrition (SEAMEO—RECFON), Jakarta, Indonesia; 5 Department of Child Health, Faculty of Medicine, Universitas Indonesia–Dr. Cipto Mangunkusumo National Central Hospital, Jakarta, Indonesia; Ateneo de Manila University Ateneo School of Medicine and Public Health, PHILIPPINES

## Abstract

**Background:**

Early initiation of breastfeeding (EIBF) is key to reducing neonatal morbidity and mortality, however, little is known about the determinants of the trends of EIBF prevalence in Indonesia. This study aims to assess the contributing factors to the changes in the prevalence of EIBF between 2007 and 2017.

**Methods:**

We analysed data from the 2007, 2012, and 2017 Indonesia Demographic and Health Surveys to estimate the trends in EIBF. A multivariate logistic decomposition model was fitted to examine variables associated with changes in the percentage of EIBF from 2007 to 2017. The contributing factors to changes in EIBF prevalence were categorized into either compositional or behavioural changes, with each of them divided into portions or percentages of contribution (pct) of the independent variables. The former refers to the changes in the distribution of samples, while the latter refers to the changes in the behavioural responses toward EIBF in both surveys. All analyses accounted for the complex study design and potential confounding factors.

**Results:**

An increase in the prevalence of EIBF from 49.9% to 56.5% was recorded between 2012 and 2017, with an overall increase of 16.9 percentage points from 2007 to 2017. At the aggregate level, the compositional differences did not significantly contribute to the changes in the percentage of EIBF, while 98.3pct (p < 0.001) was associated with changes in mothers’ behavioural response towards EIBF. The composition changes in the geographical region of Sumatra, and caesarean delivery negatively contributed to the changes in EIBF prevalence with -0.6pct and -14.2pct, respectively. However, the compositional differences in those living in Kalimantan & Sulawesi, first-time mothers, and small-born infants positively contributed to the change. Behaviour changes in mothers with higher education (8.8pct), from higher income households (-17.5pct), and those residing in Sumatra (-8.2pct) and Kalimantan & Sulawesi (-10.2pct) significantly contributed to the upward trend in EIBF prevalence.

**Conclusions:**

Almost half of the newborns experienced delayed breastfeeding initiation despite the improvement in the prevalence of EIBF. Therefore, further research and interventions on behaviour change in mother’s attitudes towards EIBF, especially among those undergoing caesarean delivery, living in Kalimantan or Sulawesi, and from wealthier households, are recommended to close this gap.

## Introduction

It has long been recognized that optimal breastfeeding practices are pivotal to maternal and child health [1, 2]. Initiating breastfeeding within the first hour after birth or early initiation of breastfeeding (EIBF) is the first recommendation of optimal breastfeeding practices, followed by exclusive breastfeeding for the first six months and continued breastfeeding with adequate complementary foods [3]. EIBF strongly predicts future exclusive and continued breastfeeding throughout infancy [4]. EIBF keep infants warmer and stimulates the production of nutrient and antibody-rich colostrum or breastmilk during the first few days of life which all facilitate bonding between mothers and their infants [5].

Besides providing all essential nutrients required for ensuring optimal growth and development of infants, EIBF has been suggested to prevent neonatal mortality by protecting infants from infection, sepsis, and acute illnesses [6–8]. Meta-analyses also demonstrated a distinct dose-response relationship in which the risk of neonatal mortality increases as breastfeeding initiation is delayed [6, 9]. Furthermore, this practice has the potential to alleviate the burden of maternal mortality through its noticeable role in reducing the risk of postpartum haemorrhage, a primary cause of this type of mortality [10]. In addition to those health benefits, economic analysis also indicates EIBF as one of the most effective interventions to prevent a child’s undernutrition, morbidity, and mortality [11].

Globally, less than half of mothers initiated breastfeeding during the first hour of life, while in Indonesia, the pooled prevalence of EIBF was 58%, or about the same as the average figure among least-developed countries [12]. The *Lancet* Breastfeeding Series 2023 also highlighted this low EIBF coverage as the practice has been lacking, particularly under the massive commercial milk formula marketing [13–15]. In Indonesia, as well as other low-and middle-income countries (LMICs), formula milk is among the most common pre-lacteal feeds [16], which is significantly correlated with delayed breastfeeding initiation [17]. Significant focus should be placed on EIBF as the first step toward successful exclusive and continued breastfeeding, especially given the current situation in the increased consumption of formula milk in Indonesia.

In terms of policy in Indonesia, exclusive breastfeeding had been introduced several years before EIBF. The government regulated exclusive breastfeeding for six months for the first time in the 2004 Minister of Health Decree on Exclusive Breastfeeding [18]. Before this regulation, the recommendation was exclusive breastfeeding for four months [19]. However, the 2004 regulation does not explicitly mention EIBF, instead endorsing health staff to help mothers breastfeed within 30 minutes after delivery. This regulation is brief and insufficient: the technical rules need to be documented, and it does not address the delegation of tasks, the authorities of implementing agencies, and the sanctions imposed for violations. In March 2012, the government explicitly included EIBF in Government Regulation No. 33/2012 on Exclusive Breastfeeding [20]. This most updated regulation adopts the current definition of EIBF and has more comprehensive aspects, such as administrative sanctions. Looking at the hierarchical order of the Indonesian legal system, the latest regulation (the Government Regulation) is at a higher level than the previous one (the Ministry of Health Decree), which indicates the government’s stronger commitment to improving breastfeeding practices [21].

Understanding the factors associated with EIBF is critical to further supports the country’s target to improve EIBF rates. Systematic reviews show that failure in EIBF is more common among first-time mothers, those undergoing caesarean deliveries, and those with low-birth-weight infants. Certain healthcare facilities may not have the necessary resources to support breastfeeding mothers of newborns with low birth weights or those undergoing caesarean deliveries. Additionally, these facilities may lack established protocols that encourage EIBF for both groups [22]. The findings outline several maternal characteristics and behaviours as essential determinants of EIBF, including parental education level, smoking status, and antenatal and postnatal check-ups as the determinants of EIBF [23, 24]. Excluding these demographic indicators, EIBF can also be influenced by socio-cultural norms that vary across regions within Indonesia [25].

The magnitude and strength of the association of variables with EIBF may depend on the contexts that are not only limited to the geographical or cultural ones but also the timeframe. Furthermore, as most studies use data at a single point, it does not allow us to observe the patterns and identify potential factors that have consistently influenced EIBF throughout different periods. Examining factors associated with changes in the prevalence of EIBF over time becomes relevant for sustainable interventions that improve EIBF rates and recommendations on breastfeeding policies and strategies. To the best of our knowledge, there are no studies in Indonesia that examine the determinants of the trends in EIBF over time. Therefore, this study aimed to investigate factors associated with the change in EIBF prevalence in Indonesia between 2007 and 2017.

## Materials and methods

### Data source, sampling procedure, and study variables

We used data from the three latest Indonesia Demographic and Health Survey (IDHS), which took place between 25^th^ of June and 31^st^ of December 2007, 7^th^ of May to 31^st^ of July 2012, and 24^th^ July and 30^th^ of September 2017 [26–28]. Thirty-three and thirty-four provinces were surveyed in 2007/2012 and 2017, respectively. Provinces are made up of districts which contain subdistricts and villages, which are then divided into urban and rural areas. The IDHS used two stages of stratified sampling, with census blocks as the primary sampling unit. The first stage is selecting census blocks using a proportional systematic random sampling method stratified by urban and rural areas, after which 25 households were systematically selected from each census block. The data were obtained from those selected households. Further information on the study design can be found in the IDHS report [28]. This study restricted the analysis to children aged 0–23 months of interviewed women aged 15–49. After removing observations with missing data, the sample size for each survey is 6,738 (2007), 6,794 (2012), and 6,568 (2017).

The outcome is EIBF, a binary variable coded ’1’ or ’Yes’ if breastfeeding was initiated within the first hour after delivery [29]. This variable is derived from the question: “How long after birth did you first put (NAME) to the breast?”. If the answer is less than one hour, the interviewer records it immediately [26–28], and we coded it as “Yes” for EIBF. The independent variables included socio-demographic variables (maternal age at delivery, education attainment, working status, father’s education, children under five in the household, nuclear family, wealth index, residence, region), infant’s and delivery factors (sex of infant, twins, perceived birth size, birth pattern, caesarean delivery, skilled birth attendant), and maternal behaviours (antenatal visit, smoking status, reading a newspaper, listening to the radio, and watching television). More information on those 20 independent variables is presented in **Table 1**.

**Table 1:** Description of the independent variables.

Variables	Definition (categories)
*Socio-demographic characteristics*	
Mother’s age at delivery	Age in years when their last child was born (15–19, 20–34, 35–49)
Mother’s education	The highest level of education attended (uncompleted primary or no formal education, completed primary, completed secondary, college or higher)
Mother currently working	If the mother worked at the time of the survey or left the job within the last 7 days (yes, no)
Father’s education	The highest level of education attended (uncompleted primary or no formal education, completed primary, completed secondary, college or higher)
Children under-five ≥2	If the total number of children under the age of five years living in the household is equal or more than two (yes, no)
Nuclear family	If the respondent lives with the nuclear family/ husband and the children (yes, no)
Wealth index	A composite index of a household’s total standard of living based on its assets, housing materials, water, and sanitation facilities. We divided the index into five equal groups or quintiles (poorest, poorer, middle, richer, richest)
Type of residency	Type of respondent’s place of residence at the time of survey/ de facto (urban, rural)
Region	The region where the respondent lives at the time of survey/ de facto. The original data are the province which then are grouped into four regions (Java & Bali, Sumatra, Kalimantan & Sulawesi, Eastern regions). The Eastern regions-group includes all provinces in Nusa Tenggara, Maluku, and Papua islands.
*Infant’s and delivery characteristics*	
Sex of infants	Sex of the infants (male, female)
Twin	If the infants are born as twins (yes, no)
Perceived birth size	Size of the infants at born, as perceived by their mother (small, average, large)
Birth pattern	A combination of birth order and preceding birth interval (first child, not first-child, birth interval <2 years, not first-child, birth interval ≥2 years)
Infant’s age	Age in months at the time of the survey
Caesarean delivery	If respondents underwent a caesarean section for their last-born children (yes, no)
Delivery at health facility	If respondents gave birth their last-born children in a health facility (yes, no)
Skilled birth attendant	If respondents gave birth their last-born children with the assistance of skilled birth attendants, such as general practitioner, obstetrician, nurse, midwife, or village midwife (yes, no)
*Maternal behaviours*	
Antenatal visits	The total number of antenatal care visits (< 4, ≥4 times)
Mother currently smoking	If respondents use any type of tobacco (yes, no)
Mother reading newspaper	If respondents read newspapers or magazines at least once a week (yes, no)
Mother listening to the radio	If respondents listen to radio at least once a week (yes, no)
Mother watching television	If respondents watch television at least once a week (yes, no)

### Statistical analysis

We used Stata/IC 15.1 for all analyses, except when obtaining values of the difference in percentages conducted in Microsoft Excel. All analyses were performed on weighted data to account for the representativeness and complex sampling procedures. We used the individual weight for women (v005) for all datasets. Descriptive statistics for respondents’ characteristics were presented as percentages by survey year. The EIBF trends were examined separately for 2007–2012, 2012–2017, and 2007–2017. The outcome’s figure by years and by each independent variable in the three surveys was also reported.

For the overall trend (2007–2017), we used a non-linear multivariate decomposition model for binary outcome using the ’mvdcmp’ command to quantify the determinants of the observed change in EIBF within the period. This approach uses a regression model to partition the difference between two groups or the shift within a particular period into covariates fitted in the model. The change or difference in the proportion between two groups can be attributed to the differences in the composition between groups (differences in characteristics) and/ or differences in the effects of independent variables (differences in coefficients) [30]. As a result, the observed increase in the prevalence of EIBF over time can be decomposed additively into a compositional difference of respondents from each survey (endowments ‘E’) component and a coefficient (or effects of characteristics) component or behavioural change ‘C’ responses for selected independent variables.

The dependent variable in a nonlinear model is a linear combination of covariates and regression coefficients:

Y=F(Xβ)=Logit(Y)=Xβ

where *Y* is the *N x* 1 dependent variable vector, *X* is an *N x K* matrix of independent variables, and *β* is a *K x* 1 vector of coefficients. The difference between group A and group B in terms of *Y* can be decomposed into:

$$Y_{A}-Y_{B}={F(X}_{A}\beta_{A})-{F(X}_{B}\beta_{B})$$

The difference in proportion in *Y* between groups A and B (in this case, group A is the 2017 survey and group B is the 2007 survey) can be decomposed as:

$$Logit(Y_{A})–Logit(Y_{B})=F(X_{A}\beta_{A})-F(X_{B}\beta_{B})$$

$$=\underset{E}{\underset{︸}{\left\{{F(X}_{A}\beta_{A})-{F(X}_{B}\beta_{A})\right\}}}+\underset{C}{\underset{︸}{\left\{{F(X}_{B}\beta_{A})-{F(X}_{B}\beta_{B})\right\}}}$$

The component labelled *E* refers to the part of the difference attributable to changes in endowments or characteristics (compositional), usually called the explained component or characteristics effect. The *C* component is the difference attributable to coefficients or behavioural change, which is usually labelled as the unexplained component. Therefore, *E* represents a counterfactual comparison of the different from the viewpoint of the data from the latest survey (2017). For example, the difference that would be anticipated if the 2017 survey were given the 2007 survey’s distribution of variables. *C* depicts a counterfactual comparison of the results from the viewpoint of the 2007 survey, representing the anticipated difference if the respondents in the 2007 survey had experienced behavioural reactions to EIBF among those in the 2017 survey. The decomposition results of *E* and *C* are at the aggregate level. To understand the contribution of each predictor in the model, we need to divide *E* and *C* into portions or percentages of contribution (Pct.), *E*_*k*_ and *C*_*k*_ (*k* = 1,…, *k*; in which *k* is the number of independent variables) [30].

To account for changes in EIBF between 2007 and 2017 in our study, the model for decomposition analysis was:

$$Logit(A)-Logit(B)=[\beta_{0A}-\beta_{0B}]+\sum \beta_{ijA}[X_{ijA}-X_{ijB}]+\sum \beta_{ijB}[\beta_{ijA}-\beta_{ijB}]$$

where:

*β*_*0A*_ is the intercept in the regression equation for IDHS 2017

*β*_*0B*_ is the intercept in the regression equation for IDHS 2007

*β*_*ijA*_ is the coefficient of the *j*^*th*^ category of the *i*^*th*^ determinant for IDHS 2017

*β*_*ijB*_ is the coefficient of the *j*^*th*^ category of the *i*^*th*^ determinant for IDHS 2007

*X*_*ijA*_ is the proportion of the *j*^*th*^ category of the *i*^*th*^ determinant for IDHS 2017

*X*_*ijB*_ is the proportion of the *j*^*th*^ category of the *i*^*th*^ determinant for IDHS 2007.

[30]

The decomposition analysis used only data from two surveys as the aim was to analyse the change in EIBF percentage between two groups (2007 and 2017). Therefore, before running the decomposition analysis, we did initial screenings for variables to be included in the decomposition model by fitting each of those into three logistic models adjusted for forced variables. Maternal education, paternal education, wealth index, region, birth pattern, perceived birth size, caesarean delivery, and place of birth were set as the forced covariates as those have been identified as important determinants of EIBF in Indonesia [31, 32] and other countries [23, 33]. These models used the samples of 2007, 2017, and both 2007 and 2017 surveys, where in the last one, the survey year was fitted as a fixed effect. All variables with *p*<0.25 in at least one logistic regression and the priory variables were included to form the final logistic models and decomposition analysis.

### Ethical considerations

The study used anonymized, secondary analysis of previously collected survey data. Before participating in the 2007, 2012, and 2017 IDHS surveys, all respondents gave their informed consent. The surveys were ethically authorized by the ICF International Review Board and the National Institute for Health and Research Development of the Indonesian Ministry of Health. Through an online request to dhsprogram.com, permission to access the IDHS datasets was obtained for this study.

## Results

### Respondents’ characteristics

Just over 70% of the respondents were at the reproductive ages (20–34 years) when giving birth to their last-born in the three surveys (**Table 2**). During the ten years, mothers who completed secondary and higher education increased by 7.1% and 8.3%, respectively. At the same time, it saw a positive trend for delivery at health facilities, delivery assisted by skilled birth attendants, and antenatal visits, which recorded percentage point (pp) changes at 33pp, 15.3pp, and 8.6pp, respectively. The percentage of caesarean delivery doubled to almost 20%, while that of small-born infants slightly decreased to 11.8%.

**Table 2:** Percentage distribution of the respondents’ characteristics in 2007, 2012, and 2017 Indonesian Demographic and Health Surveys and the percentage point differences.

Characteristics	2007 (%)	2012 (%)	2017 (%)	Percentage point difference (pp)		
	N = 6,738	N = 6,794	N = 6,568	2012–2007	2017–2012	2017–2007
Mother’s age at delivery (years)						
≤19	12.3	12.3	8.7	0.0	-3.6	-3.6
20–34	74.1	73.2	73.8	-0.9	0.6	-0.2^1^
≥35	13.7	14.5	17.5	0.9^1^	2.9^1^	3.8
Mother’s education						
Uncompleted primary or no formal education	12.1	8.7	6.0	-3.4	-2.7	-6.1
Completed primary	55.0	49.6	45.7	-5.5^1^	-3.8^1^	-9.3
Completed secondary	24.1	28.4	31.2	4.2^1^	2.8	7.1
College or higher	8.8	13.4	17.1	4.6	3.7	8.3
Mother currently working, yes	37.8	39.1	36.8	1.3	-2.3	-1.0
Father’s education						
Uncompleted primary or no formal education	12.1	8.9	6.6	-3.2	-2.4^1^	-5.6
Completed primary	48.0	47.6	43.3	-0.4	-4.3	-4.7
Completed secondary	30.8	31.1	35.3	0.4^1^	4.2	4.5
College or higher	9.2	12.4	14.9	3.2	2.5	5.7
Children under-five ≥2, yes	5.5	4.5	4.3	-1.0	-0.2	-1.2
Nuclear family, yes	68.7	61.4	61.8	-7.3	0.4	-6.9
Wealth index						
Poorest	20.2	19.5	19.2	-0.7	-0.3	-1.1^1^
Poorer	18.3	21.0	20.2	2.7	-0.9^1^	1.9
Middle	21.0	19.9	19.8	-1.2^1^	0.0^1^	-1.2
Richer	20.8	20.6	21.3	-0.2	0.8^1^	0.5
Richest	19.7	19.1	19.5	-0.6	0.5^1^	-0.2
Type of residency, urban	42.7	49.9	49.4	7.2	-0.5	6.7
Region						
Java & Bali	56.0	56.6	56.3	0.5^1^	-0.3	0.3
Sumatra	22.5	22.2	23.1	-0.3	0.9	0.6
Kalimantan & Sulawesi	14.5	14.0	13.1	-0.4^1^	-1.0^1^	-1.4
Eastern region	7.0	7.2	7.6	0.2	0.3	0.5^1^
Sex of infants, female	46.8	48.9	48.2	2.1	-0.7	1.4
Twins, yes	0.8	0.8	0.7	-0.1	-0.1	-0.2
Perceived birth size						
Small	14.2	13.8	11.8	-0.4	-2.0	-2.4
Average	53.1	56.5	57.2	3.5^1^	0.6^1^	4.1
Large	32.7	29.7	31.1	-3.1^1^	1.4	-1.7^1^
Birth pattern						
First child	36.5	39.4	32.7	2.9	-6.7	-3.8
Not first-child, birth interval <2 years	7.8	6.0	5.5	-1.8	-0.4^1^	-2.2^1^
Not first-child, birth interval ≥2 years	55.7	54.7	61.8	-1.1^1^	7.2^1^	6.1
Caesarean delivery, yes	8.5	14.0	19.6	5.4^1^	5.6	11.1
Delivery at health facility, yes	51.5	70.5	84.4	19.0	14.0^1^	33.0^1^
Skilled birth attendant, yes	78.3	87.4	93.6	9.1	6.2	15.3
Antenatal visits, ≥4	82.5	88.9	91.1	6.5^1^	2.2	8.6
Mother currently smoking, yes	1.3	1.9	1.4	0.6	-0.5	0.1
Mother reading newspaper, yes	12.8	11.3	8.62	-1.5	-2.7	-4.2
Mother listening to the radio, yes	26.8	15.9	10.8	-10.8	-5.2	-16.1^1^
Mother watching television, yes	77.7	84.6	83.7	6.9	-0.8^1^	6.1^1^

^1^Differences are due to rounding

### Trends of early initiation of breastfeeding

**Fig 1:**
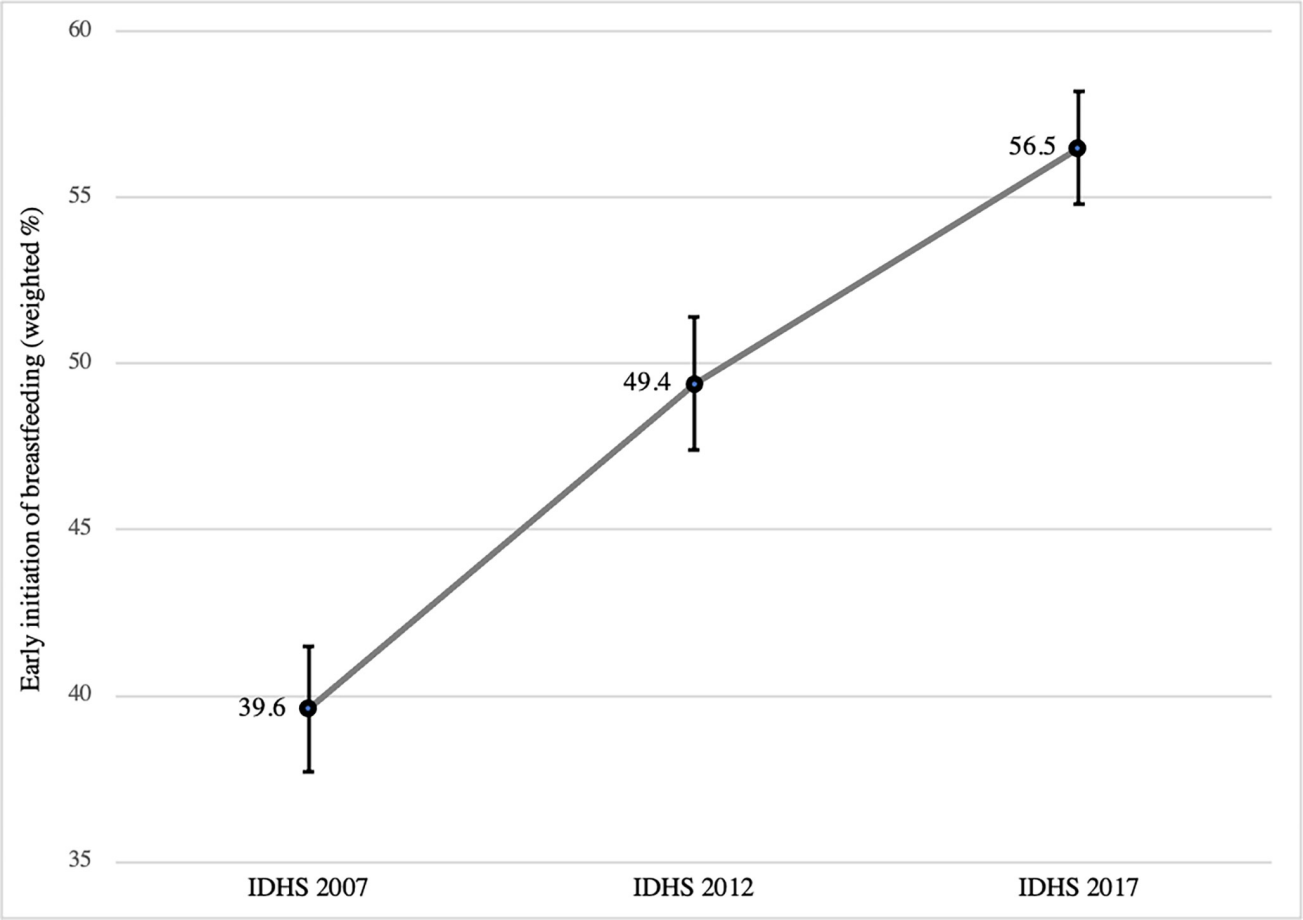
Trends of early initiation of breastfeeding in Indonesia, 2007–2017.

**Fig 2:**
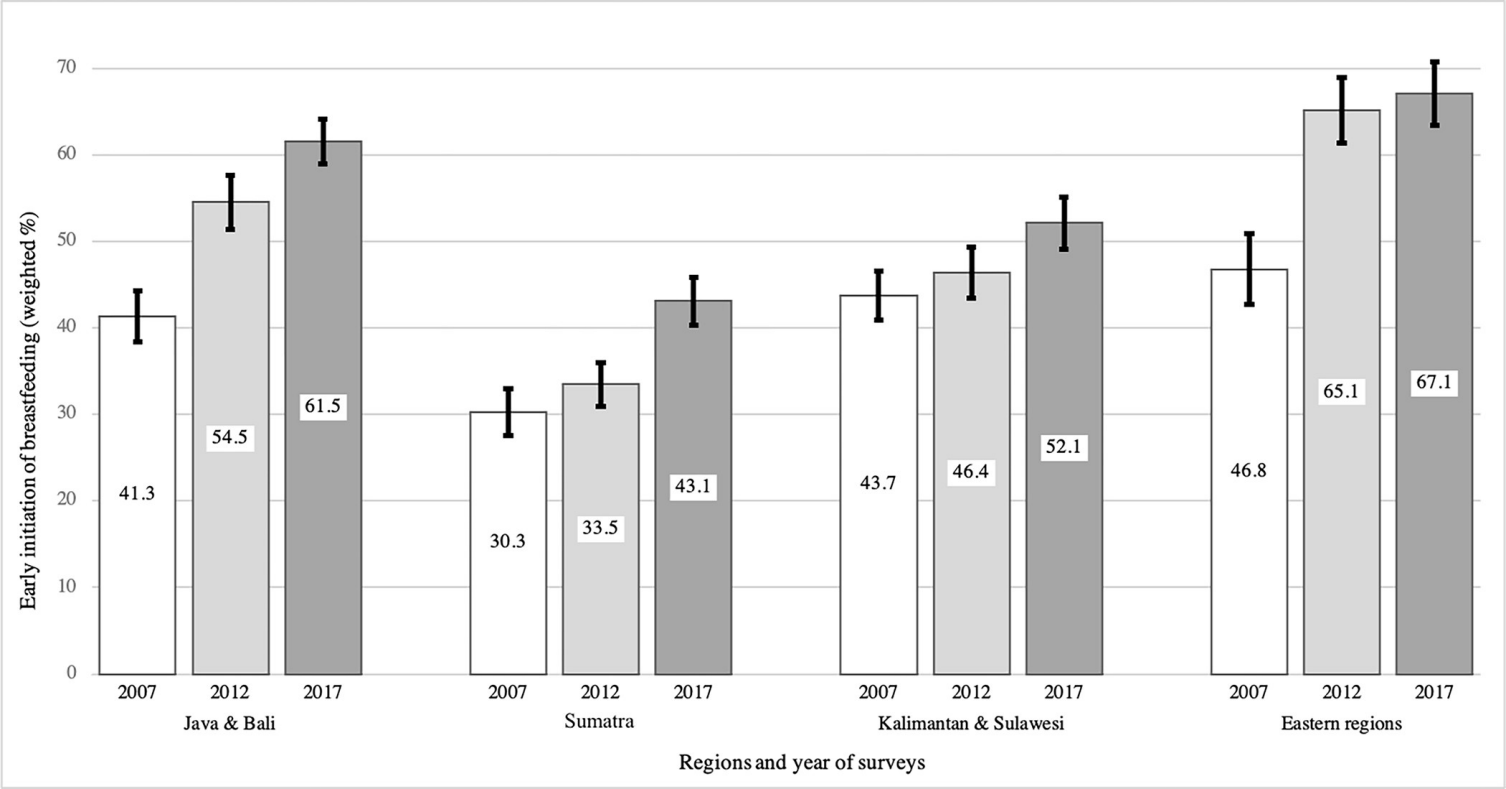
Trends of early initiation of breastfeeding in Indonesia by regions, 2007–2017.

We divided the EIBF trends into three phases: 2007–2012, 2012–2017, and 2007–2017 respectively. Overall, EIBF across the country increased by 16.9pp from 39.6% (min–max across regions = 37.7%– 41.5%) to 56.5% (54.8%– 58.2%) between 2007 and 2017, with an average increase of 1.7pp annually (**Fig 1**). The results also showed that the progress made between 2007 and 2012 was higher at 9pp than the second recording period between 2012 and 2017 at 7pp, showing a decline in the uptake of EIBF over time. By regions, we can see that from 2007 to 2017, Java & Bali and other Eastern regions saw the biggest progress in EIBF, just over 20pp each (**Fig 2**). Concurrently, EIBF in Kalimantan and Sulawesi only improved by 8.4pp or 5.5pp, respectively, lower than the increase in Sumatra.

**Table 3** presents the trends by respondents’ characteristics. In each category of the variables, the majority of results show positive changes, but EIBF prevalence decreased by almost 7pp between 2007–2012 among smoking mothers. A smaller negative trend can be seen among respondents whose husbands did not finish primary school from 2012–2017, while among those attending college, the prevalence of EIBF improved across the study period. By wealth index, the most considerable progress was among respondents at the middle socioeconomic level.

**Table 3:** Trends in the percentage of early initiation of breastfeeding by respondents’ characteristics between 2007 and 2017 Indonesia demographic and health surveys.

Characteristics	2007	2012	2017	Percentage point difference		
	N = 6,738	N = 6,794	N = 6,568	2012–2007	2017–2012	2017–2007
Mother’s age at delivery (years)						
≤19	35.9	49.3	54.8	13.4	5.5	18.9
20–34	40.3	49.1	56.7	8.9	7.6	16.5^1^
≥35	39.1	51.2	56.1	12.1	4.9	17.0
Mother’s education						
Uncompleted primary or no formal education	46.3	53.8	54.2	7.5	0.4	7.9
Completed primary	40.8	51.0	59.0	10.3^1^	7.9^1^	18.2
Completed secondary	36.0	47.0	53.7	11.0	6.7	17.7
College or higher	32.7	45.9	55.7	13.2	9.8	23.0
Mother currently working, yes	39.0	48.2	55.5	9.2	7.4^1^	16.5
Father’s education						
Uncompleted primary or no formal education	41.6	55.0	53.1	13.5^1^	-1.9	11.6^1^
Completed primary	39.9	50.6	57.6	10.7	7.0	17.7
Completed secondary	39.9	47.1	55.7	7.2	8.6	15.8
College or higher	34.1	46.9	56.5	12.8	9.6	22.4
Children under-five ≥2, yes	36.7	53.0	58.3	16.4^1^	5.3	21.6
Nuclear family, yes	41.1	50.5	58.2	9.4	7.7	17.1
Wealth index						
Poorest	44.3	53.2	58.1	8.9	4.9	13.8
Poorer	41.1	49.4	57.4	8.3	8.0	16.3
Middle	34.1	47.3	57.4	13.3^1^	10.0^1^	23.3
Richer	42.4	49.1	52.5	6.7	3.5^1^	10.2^1^
Richest	36.2	48.2	57.2	12.0	9.0	21.0
Type of residency, urban	37.9	48.7	57.0	10.8	8.3	19.0^1^
Sex of infants, female	38.3	49.6	57.3	11.4^1^	7.7	19.0
Twin, yes	25.3	32.6	41.7	7.2	9.2^1^	16.4
Perceived birth size						
Small	35.6	45.9	46.9	10.4	1.0	11.4^1^
Average	41.1	49.9	58.3	8.8	8.4	17.2
Large	38.9	50.2	56.7	11.3	6.5	17.8
Birth pattern						
First child	33.8	43.3	49.9	9.5	6.6	16.2^1^
Not first-child, birth interval <2 years	41.2	54.1	59.1	12.9	5.0	17.9
Not first-child, birth interval ≥2 years	43.2	53.4	59.7	10.2	6.3	16.5
Caesarean delivery, yes	25.0	31.5	37.0	6.5	5.5	12.0
Delivery at health facility, yes	38.6	49.3	56.6	10.7	7.3	18.0
Skilled birth attendant, yes	39.1	48.7	56.5	9.6	7.9^1^	17.4
Antenatal visits, ≥4	39.6	49.9	57.0	10.3	7.1	17.4
Mother currently smoking, yes	52.6	45.8	54.1	-6.9^1^	8.4^1^	1.5
Mother reading newspaper, yes	38.4	48.0	53.2	9.6	5.1^1^	14.8
Mother listening to the radio, yes	35.6	48.8	54.0	13.2	5.2	18.4
Mother watching television, yes	38.1	48.4	56.3	10.3	7.9	18.2
Region						
Java & Bali	41.3	54.5	61.5	13.2	7.0	20.2
Sumatra	30.3	33.5	43.1	3.2	9.7^1^	12.8
Kalimantan & Sulawesi	43.7	46.4	52.1	2.6^1^	5.8^1^	8.4
Eastern region	46.8	65.1	67.1	18.3	2.0	20.3

^1^Differences are due to rounding

### Factors associated with the progress in early initiation of breastfeeding

**Table 4** presents the results of the decomposition analysis at the aggregate level for the changes due to differences in compositional factors or characteristics (*E*) and those due to differences in coefficients or behavioural (*C*), as well as the contribution of each predictor to the overall component of *E* and *C*. Results indicated that, after controlling the roles of composition factors, around 98% of the increase in the EIBF prevalence from 2007 to 2017 was due to behavioural changes that positively impact EIBF. However, the aggregate contribution of the changes in compositional factors towards the upward trend of EIBF was not statistically significant after adjusting for behavioural changes. The contribution of each predictor estimated by dividing *E* and *C* into portions (or percentage of contribution) are explained in the subsequent two paragraphs.

**Table 4:** Results of multivariate decomposition analysis for the change in the percentage of early initiation of breastfeeding in Indonesia, 2007–2017.

Characteristics	Differences due to compositional factors (*E*)					Differences due to behavioral factors (*C*)				
	Coeff.	95% CI		Pct.		Coeff.	95% CI		Pct.	
Mother’s age at delivery (years)										
≤19	-0.0018	-0.0040	0.0004	-1.09		0.0074	-0.0031	0.0179	4.38	
20–34	Ref.					Ref.				
≥35	-0.0007	-0.0021	0.0008	-0.40		0.0047	-0.0051	0.0146	2.81	
Mother’s education										
Uncompleted primary or no formal education	Ref.					Ref.				
Completed primary	-0.0020	-0.0080	0.0040	-1.17		0.0400	-0.0101	0.0901	23.68	
Completed secondary	0.0009	-0.0039	0.0057	0.53		0.0247	-0.0002	0.0495	14.61	
College or higher	0.0054	-0.0021	0.0129	3.17		0.0149	0.0029	0.0267	8.81	*
Father’s education										
Uncompleted primary or no formal education	Ref.					Ref.				
Completed primary	-0.0018	-0.0047	0.0011	-1.05		0.0085	-0.0344	0.0515	5.05	
Completed secondary	0.0030	-0.0005	0.0064	1.75		0.0043	-0.0261	0.0346	2.54	
College or higher	0.0037	-0.0012	0.0086	2.19		0.0026	-0.0091	0.0143	1.55	
Children under-five ≥2, yes	-0.0002	-0.0010	0.0005	-0.13		0.0035	-0.0020	0.0090	2.05	
Nuclear family, yes	-0.0019	-0.0042	0.0003	-1.13		0.0021	-0.0340	0.0412	1.25	
Wealth index										
Poorest	-0.0002	-0.0007	0.0003	-0.12		-0.0150	-0.0311	0.0011	-8.89	
Poorer	0.0001	-0.0007	0.0010	0.07		-0.0127	-0.0269	0.0015	-7.52	
Middle	Ref.					Ref.				
Richer	-0.0003	-0.0006	0.0000	-0.16		-0.0295	-0.0458	-0.0135	-17.49	***
Richest	0.0000	-0.0001	0.0001	0.01		-0.0082	-0.0259	0.0096	-4.83	
Region										
Java & Bali	Ref.					Ref.				
Sumatra	-0.0011	-0.0018	-0.0004	-0.63	**	-0.0138	-0.0273	-0.0004	-8.20	*
Kalimantan & Sulawesi	0.0014	0.0004	0.0024	0.83	**	-0.0173	-0.0263	-0.0083	-10.24	***
Eastern region	0.0001	-0.0002	0.0004	0.06		0.0001	-0.0050	0.0053	0.07	
Sex of infants, female	0.0001	-0.0003	0.0005	0.06		0.0164	-0.0046	0.0373	9.69	
Perceived birth size										
Small	0.0019	0.0003	0.0035	1.13	*	-0.0029	-0.0131	0.0073	-1.71	
Average	Ref.					Ref.				
Large	0.0002	-0.0003	0.0007	0.12		0.0034	-0.0147	0.0214	1.98	
Birth pattern										
First child	0.0039	0.0009	0.0069	2.30	*	0.0085	-0.0310	0.0140	-5.04	
Not first-child, birth interval <2 years	0.0001	-0.0012	0.0013	0.05		0.0002	-0.0068	0.0072	0.10	
Not first-child, birth interval ≥2 years	Ref.					Ref.				
Caesarean delivery, yes	-0.0241	-0.0398	-0.0083	-14.25	**	-0.0067	-0.0139	0.0004	-3.99	
Delivery at health facility, yes	0.0028	-0.0135	0.0191	1.65		-0.0069	-0.0430	0.0292	-4.08	
Skilled birth attendant, yes	0.0075	-0.0048	0.0198	4.46		0.0229	-0.0497	0.0955	13.53	
Antenatal visits, ≥4	0.0035	-0.0009	0.0079	2.08		0.0093	-0.0491	0.0677	5.53	
Mother currently smoking, yes	-0.0000	-0.0001	0.0001	-0.02		-0.0022	-0.0049	0.0005	-1.30	
Mother listening to the radio, yes	0.0022	-0.0054	0.0098	1.30		0.0067	-0.0106	0.0239	3.95	
Mother watching television, yes	0.0002	-0.0022	0.0025	0.09		0.0355	-0.0138	0.0848	21.03	
*Constants*						*0*.*0827*	*-0*.*0850*	*0*.*2505*	*48*.*97*	
*Overall*	*0*.*0029*	*-0*.*0146*	*0*.*0203*	*1*.*69*		*0*.*1661*	*0*.*1359*	*0*.*1962*	*98*.*31*	***
*Residual*	*0*.*1689*	*0*.*1450*	***							

Coeff., Decomposition coefficients; CI, Confidence Interval; Pct., Percentage contribution of each variable category to the overall increase in early initiation of breastfeeding from 2007 to 2017 in Indonesia; Ref., Reference.

***p<0.001

**p<0.01

*p<0.05

Among independent variables contributing to the increase in EIBF prevalence, differences in the percentage of caesarean delivery explained the majority of the differences due to compositional factors or *E* with -14.2% of contribution (pct) and *E* coefficient (*E*_*coeff*_) -0.0241. The negative *E*_*coeff*_ indicated the expected *decrease* if the percentage of caesarean delivery in the 2017 IDHS was the same as in 2007. The figure for caesarean delivery increased from 8.5% in 2007 to 19.6% in 2017 (**Table 2**). Differences in the compositional factors of regions, birth patterns, and perceived birth weight also contributed to the change in EIBF prevalence. Further, compared to Java & Bali, a small increase in the percentage of mothers living in Sumatra during the study period was associated with the change in EIBF with -0.6pct, similar to the changes in caesarean delivery. However, a decreased percentage of those living in Kalimantan & Sulawesi showed a positive contribution (*E*_*coeff*_ 0.0014; 0.8pct). Regarding birth pattern, the change in the prevalence of first-time mothers—from 36.5% in 2007 to 32.7% in 2017 contributed to 2.3pct in the change in EIBF. The percentage of small-born infants as perceived by the mothers, declined from 14.2% in 2007 to 11.8% in 2017. This change in the composition of the population also yielded a notably modest favourable effect on the progress in the percentage of EIBF.

By controlling all the compositional change factors, the percentage of EIBF increased due to behavioural changes toward EIBF among mothers with college or higher education, higher income category of the wealth index, and those living in Sumatra or Kalimantan & Sulawesi. The biggest contribution came from the behavioural change among the wealthier group, with *C* coefficient (*C*_*coeff*_) and -17.5pct. The result indicated that the behavioural changes among that group of mothers negatively affected the prevalence of EIBF. Behavioural changes among mothers from Sumatra or Kalimantan & Sulawesi also indicated the same trend, with -8.2pct and -10.2pct, respectively. This means the *increase* in EIBF would have been around 20% higher if the responses among both groups towards EIBF had not changed within the period. In contrast, we found behavioural changes toward EIBF among mothers who attended college or higher education positively contributed to the observed change in the percentage of EIBF (*C*_*coeff*_ 0.0149; 8.8pct).

## Discussion

This study examined the trends of EIBF in 2007, 2012, and 2017 and decomposed factors that contributed to changes in EIBF from 2007 to 2017. To the best of our knowledge, this is the first study to analyse the determinants of the change in EIBF in Indonesia using the multivariate decomposition method. This study is increasingly relevant given the changes in Indonesia’s policies on breastfeeding, particularly EIBF. Although the prevalence of EIBF improved sustainably from 2007 to 2017, it remains low with more than 4 in 10 infants not put on their mother’s breast in the first hour of life. This figure is slightly higher than the global average of 47%, but more efforts should be made to achieve the 2030 global target of 70% [34]. Growing support for EIBF in the national law on health, particularly after 2012, may partly explain the observed change.

The multivariate decomposition analysis demonstrated that the contribution of behavioural changes was greater than that of changes in the distribution of respondents by characteristics to the positive trend in the percentage of EIBF in Indonesia from 2007 to 2017. Adjusting the role of changes in behaviours, the overall contribution of the compositional factor was not statistically significant. However, by variables, there was strong evidence that the changes in the percentage of caesarean delivery, birth pattern, perceived birth size, and regions contributed to the shift in EIBF. We also found that after keeping the compositional changes constant, behaviour towards EIBF among mothers with higher education positively contributed to the increase in EIBF prevalence. This may be the result of increased information accessibility, enabling women with greater education to utilise it more effectively than those with less education. In contrast, the behavioural changes among respondents living in Sumatra or Kalimantan and those from a wealthier family recorded negative contributions. In some cultures, formula feeding is associated with wealth and modernity [35]. This phenomenon is accompanied by commercial milk formula marketing that is rapidly growing, which may explain why mothers, particularly from the wealthier group, disregard EIBF [14].

Caesarean delivery has been widely accepted as a critical barrier to EIBF in almost all countries, including Indonesia [23, 32, 33, 36, 37]. An increase of almost 20% (2017) in the rate of caesarean deliveries had a negative contribution to the change in EIBF. In 2007, due to a lack of medical access, the rate of caesarean delivery was less than 10%. However, within a decade, that rate increased to almost 20%, or above the most optimal rate of 15% recommended by the WHO [38]. Similar situations exist globally, as the prevalence nearly doubled from 12.1% in 2000 to 21.1% in 2015 [38]. Our decomposition analysis showed an expected additional increase in the EIBF growth if the percentage of caesarean delivery in 2017 was at the same level as in the previous ten years. This result was in line with the decomposition analysis from Ethiopia [39]. Further, as the number of women having caesarean deliveries keeps increasing globally and in Indonesia, our findings become more concerning [38]. Although the percentage is generally lower in LMICs, we should note that caesarean delivery went up rapidly in LMICs, particularly among higher-income groups and those living in urban areas [40, 41]. There is typically a higher financial compensation for medical professionals and healthcare facilities in the case of a caesarean section compared to a vaginal delivery [42]. This disparity in payment may potentially contribute to the prevalence of non-indicated caesarean sections. Additionally, access to biased information can affect women’s preference for caesarean sections, and those from higher socioeconomic backgrounds and urban areas are more likely to undergo the procedure [41]. Therefore, interventions reducing medically unnecessary caesarean deliveries should be improved. These interventions may include financial and regulatory strategies, as well as educational initiatives to enhance women’s understanding of caesarean sections [42]. Our decomposition analysis also indicated that the behaviours of mothers undergoing caesarean deliveries in 2007 and 2017 were not associated with the changes in the rate of EIBF. This suggests that interventions should focus on improving the behaviours of these mothers towards EIBF, such as, enhancing the standard operation procedure on EIBF after caesarean deliveries through collaborative actions among staff members in hospitals [43] and discouraging the prevalent practice of separating mothers and newborns after caesarean sections [38].

Previous studies show that first-time mothers are less likely to adopt EIBF [44]. Therefore, if the proportion of this group decreases, we will expect an increase in the EIBF prevalence after controlling for other variables. Our analysis aligns with that conclusion: the downward trend in the percentage of first-time mothers within a decade positively contributed to the change in EIBF. Previous decomposition analysis in Ethiopia also has a similar result: increasing the number of infants of second and higher birth order positively contributes to the increase in EIBF [39]. Besides not having breastfeeding experience, first-time mothers are less likely to have adequate knowledge of EIBF and general breastfeeding practices, which influences their attitudes, confidence and decision to adopt this practice [45]. This phenomenon is consistent with our findings on the significant contribution of the changes in the proportion of first-time mothers in the increased percentage of EIBF from 2007 to 2017. Further, first-time mothers are generally younger than those having second or more children. This variable is closely related to the age of first marriage, which slightly increased over the decade [26, 28]. The age of first-time mothers increased in 2017 when combined with the advancement of technology and access to information, this group may have had increased knowledge on EIBF which may have accounted for positive changes int he prevalence between 2007 and 2017. However, this unobservable behavioural change did not significantly contribute to the progress in the prevalence of EIBF after adjusting for the compositional differences. The result could indicate that the positive behavioural changes among first-time mothers within that period were inadequate to boost the improvement in the EIBF rate; therefore, more strategic interventions are needed for this group. For example, improving access to support from professionals and partners (family members) leads to positive attitudes toward EIBF [46, 47]. The downward trend in fertility [48], meaning that the proportion of first-time mothers might be bigger than those with more than one child, makes EIBF interventions targeted to this group of mothers more crucial than ever.

Fewer infants perceived as small at birth in the last survey contributed to the progress of EIBF prevalence in Indonesia. The result also aligned with a previous analysis of LMIC settings [39]. Determinants analyses also conclude that EIBF is less common among infants who are smaller than average as perceived by mothers [32, 49, 50], as these infants may have specific conditions, such as an illness, that require them to be separated from the mothers for necessary treatments in the Neonatal Intensive Care Unit (NICU). An area for improvement also includes the hospital policy that continues to separate mothers and infants after birth, even when both are in good health [51]. Certain hospitals have a policy that permits rooming-in exclusively for mothers who cover the costs of their inpatient stay through out-of-pocket expenses or private insurance. Consequently, individuals lacking the financial ability to obtain private insurance face a disadvantage in accessing rooming-in, a crucial element of the ten steps for successful breastfeeding in the Baby-Friendly Hospital Initiative (BFHI). The fourth step of this initiative emphasizes the importance of EIBF. In addition, infants being regarded as small by their mothers may imply that newborns have low birth weight, which is one of the barriers to EIBF outlined in a systematic review [36]. Kangaroo mother care (KMC), a practice involving skin-to-skin contact between parents and their infants, can be performed to ensure better breastfeeding outcomes, particularly for low birth weight and/or preterm infants. However, its implementation remains limited in Indonesian hospitals [52]. Therefore, mothers in this group should be prioritized on interventions, such as promotions on the importance of EIBF and training for health staff to provide support in helping mothers with EIBF. This study also found no significant contribution to the change in the effects or behaviours among mothers of small-born infants in both surveys. In other words, the behaviours of those mothers toward EIBF might not change, which indicates opportunities to direct the behaviours in more positive ways through the interventions.

By region, the figures of EIBF were not equal across the time frame. However, we can see that Java and Bali had similar EIBF prevalence to the Eastern regions. Further, the changes indicated the same pattern: both regions experienced similar growth. The decomposition analysis results confirmed that by fitting Java and Bali as the reference group, the changes in the Eastern regions, either by the compositions or the effects, had no contribution to the shift in EIBF as the prevalence in both regions was not different. Previous analyses of earlier surveys also showed that the prevalence of EIBF in Java & Bali and the Eastern parts of the country did not significantly differ, and EIBF was less common among mothers in Sumatra [53].

Significant negative contributions were found among respondents from Sumatra and Kalimantan due to the changes in behaviours towards EIBF. As a result, if the percentage increases, we will expect a negative contribution, due to the composition change, and vice versa. The adverse effects of behaviour changes toward EIBF among mothers in both regions may be attributed to the differences in policies and programs related to EIBF. Indonesia has implemented a system of regional autonomy, wherein each region possesses the rights, authorities, and obligations to govern and oversee their respective local communities, including when it comes to health matters. Certain provinces and regencies have introduced legal provisions to facilitate the implementation of this practice. As seen in Klaten Regency in Central Java where explicit regulations have been established for EIBF since 2008, which was subsequently supplemented by further legislation in 2018 and revised in 2019 [54]. Another explanation could be cultural differences relevant to breastfeeding practices. For example, there is a fundamental philosophy in the Bajo culture of Sulawesi stating that skin-to-skin contact, which fosters EIBF significantly, establishes lifelong bonds between mothers and infants [55].

The compositional difference in maternal education did not contribute to the change in the prevalence of EIBF. Nonetheless, there was evidence that behavioural shifts among mothers with higher education increased EIBF practice. This finding aligned with systematic reviews/meta-analyses showing that more educated mothers tend to perform EIBF [23, 36]. However, decomposition analysis of the 2005 and 2016 Ethiopian DHS finds maternal education is not among the contributing factors [39]. The difference might be attributed to variations in methodologies, particularly in which maternal education categories are aggregated and the contextual circumstances in each respective country.

Behavioural changes among mothers from higher-income families had the biggest contribution to the shift in the prevalence of EIBF. Studies in several Asian countries also indicate that EIBF was more common in lower socioeconomic backgrounds [49]. In Indonesia, this finding might be attributed to breastfeeding interventions or programs that focused more on households with lower incomes. In 2007, the government introduced the Family Hope Program, a conditional cash transfer initiative designed to assist low-income and vulnerable households, in which health check-ups for pregnant and lactating women are among the conditions. The impact evaluations report positive effects on health-seeking behaviours, mothers’ knowledge and practice of breastfeeding, and stunting reduction [56]. Another possible explanation could be related to the influence of marketing strategies for breastmilk substitutes, which may be particularly relevant for higher-income families since they have a higher capacity to purchase such products. Simultaneously, mothers within this group may perceive breastfeeding as a choice of infant feeding that can be substituted by alternatives, such as infant formula, seen as a characteristic of contemporary society [57]. The industry of breastmilk substitutes has been rapidly growing, with Indonesia emerging as a prominent market. Additionally, it is worth noting that the government has yet to fully adopt the International Code of Marketing of Breast Milk Substitutes, which protects mothers against the industry’s aggressive marketing tactics, which potentially shape their perceptions regarding recommended breastfeeding practices, including EIBF [58].

Among the strengths of the present analysis is using data from standardized national surveys, the 2007–2017 IDHS, which means the conclusions can be generalized for the Indonesian population. The analysis employing statistical methods with appropriate adjustments—for complex study design and confounding factors—resulted in more reliable estimates. However, recall bias might occur as some information happened sometime before the data collection. Lastly, the survey did not cover information on other important variables related to EIBF, such as mothers’ attitudes towards cultural or local beliefs related to EIBF and the availability of early breastfeeding support.

## Conclusion

Over a period of ten years, Indonesia experienced a gradual increase in the prevalence of EIBF, however, the rate of increase slowed down. Several variables were identified as contributors to the growth. This study had several recommendations to continue this positive trend or even to speed it up to achieve the global target on EIBF. Further research assessing unobserved behaviours towards EIBF, especially among mothers from wealthier families, undergoing caesarean delivery, and living in Kalimantan & Sulawesi, would give a deeper understanding of this topic and bring insights for more effective interventions. For instance, by addressing the overuse of caesarean section [38] and, at the same time, providing breastfeeding support for mothers undergoing that procedure [47]. Lastly, it is also crucial to continue and scale up the existing programs related to EIBF and strengthen the implementation of the relevant regulations.

## Supporting information

S1 File STROBE checklist. (PDF)

## References

[1] Jones G, Steketee RW, Black RE, Bhutta ZA, Morris SS. How many child deaths can we prevent this year? The Lancet. 2003;362: 65–71. doi: 10.1016/S0140-6736(03)13811-1 12853204

[2] Victora CG, Bahl R, Barros AJD, França GVA, Horton S, Krasevec J, et al. Breastfeeding in the 21st century: epidemiology, mechanisms, and lifelong effect. Lancet Lond Engl. 2016;387: 475–490. doi: 10.1016/S0140-6736(15)01024-7 26869575

[3] World Health Organization and the United Nations Children’s Fund (UNICEF), UNICEF, editors. Global strategy for infant and young child feeding. Geneva: WHO; 2003. Available: https://www.who.int/publications/i/item/9241562218

[4] Permatasari TAE, Syafruddin A. Early Initiation of Breastfeeding Related to Exclusive Breastfeeding and Breastfeeding Duration in Rural and Urban Areas in Subang, West Java, Indonesia. 5. 2016;30: Journal of Health Research. doi: 10.14456/JHR.2016.46

[5] World Health Organization, United Nations Children’s Fund (UNICEF). Implementation guidance: protecting, promoting and supporting breastfeeding in facilities providing maternity and newborn services: the revised baby-friendly hospital initiative. Geneva: World Health Organization; 2018. Available: https://apps.who.int/iris/handle/10665/272943

[6] Khan J, Vesel L, Bahl R, Martines JC. Timing of Breastfeeding Initiation and Exclusivity of Breastfeeding During the First Month of Life: Effects on Neonatal Mortality and Morbidity—A Systematic Review and Meta-analysis. Matern Child Health J. 2015;19: 468–479. doi: 10.1007/s10995-014-1526-8 24894730

[7] Raihana S, Dibley MJ, Rahman MM, Tahsina T, Siddique MdAB, Rahman QS, et al. Early initiation of breastfeeding and severe illness in the early newborn period: An observational study in rural Bangladesh. Rasmussen K, editor. PLOS Med. 2019;16: e1002904. doi: 10.1371/journal.pmed.1002904 31469827 PMC6716628

[8] Debes AK, Kohli A, Walker N, Edmond K, Mullany LC. Time to initiation of breastfeeding and neonatal mortality and morbidity: a systematic review. BMC Public Health. 2013;13 Suppl 3: S19. doi: 10.1186/1471-2458-13-S3-S19 24564770 PMC3847227

[9] Smith ER, Hurt L, Chowdhury R, Sinha B, Fawzi W, Edmond KM, et al. Delayed breastfeeding initiation and infant survival: A systematic review and meta-analysis. Simeoni U, editor. PLOS ONE. 2017;12: e0180722. doi: 10.1371/journal.pone.0180722 28746353 PMC5528898

[10] Saxton A, Fahy K, Rolfe M, Skinner V, Hastie C. Does skin-to-skin contact and breast feeding at birth affect the rate of primary postpartum haemorrhage: Results of a cohort study. Midwifery. 2015;31: 1110–1117. doi: 10.1016/j.midw.2015.07.008 26277824

[11] Bhutta ZA, Ahmed T, Black RE, Cousens S, Dewey K, Giugliani E, et al. What works? Interventions for maternal and child undernutrition and survival. Lancet Lond Engl. 2008;371: 417–440. doi: 10.1016/S0140-6736(07)61693-6 18206226

[12] UNICEF. On my mind: promoting, protecting and caring for children’s mental health. New York, NY: UNICEF; 2021. Available: https://www.unicef.org/reports/state-worlds-children-2021

[13] Pérez-Escamilla R, Tomori C, Hernández-Cordero S, Baker P, Barros AJD, Bégin F, et al. Breastfeeding: crucially important, but increasingly challenged in a market-driven world. The Lancet. 2023;401: 472–485. doi: 10.1016/S0140-6736(22)01932-8 36764313

[14] Rollins N, Piwoz E, Baker P, Kingston G, Mabaso KM, McCoy D, et al. Marketing of commercial milk formula: a system to capture parents, communities, science, and policy. The Lancet. 2023;401: 486–502. doi: 10.1016/S0140-6736(22)01931-6 36764314

[15] Baker P, Smith JP, Garde A, Grummer-Strawn LM, Wood B, Sen G, et al. The political economy of infant and young child feeding: confronting corporate power, overcoming structural barriers, and accelerating progress. The Lancet. 2023;401: 503–524. doi: 10.1016/S0140-6736(22)01933-X 36764315

[16] Nurokhmah S, Masitoh S, Werdani KE. Prevalence and Determinants of Pre-lacteal Feeding: Insights from the 2017 Indonesia Demographic and Health Survey. Kesmas Natl Public Health J. 2021;16. doi: 10.21109/kesmas.v16i2.4283

[17] Pérez-Escamilla R, Hromi-Fiedler A, Rhodes EC, Neves PAR, Vaz J, Vilar-Compte M, et al. Impact of prelacteal feeds and neonatal introduction of breast milk substitutes on breastfeeding outcomes: A systematic review and meta-analysis. Matern Child Nutr. 2022;18 Suppl 3: e13368. doi: 10.1111/mcn.13368 35489107 PMC9113480

[18] Ministry of Health (Kemenkes). Decree of the Minister of Health of the Republic of Indonesia No 450/MENKES/SK/VI/2004 on Exclusive Breastfeeding in Indonesia. 2004.

[19] Ministry of Health (Kemenkes). Decree of the Minister of Health of the Republic of Indonesia No. 237/Menkes/SK/IV/1997 on Marketing of Breast Milk Substitutes. 1997.

[20] Government of the Republic Indonesia. Government Regulation No. 33/2012 on Exclusive Breastfeeding. 2012.

[21] ASEAN Law Association. Legal System. Legal System in Indonesia 2005. Singapore: ASEAN Law Association; 2019. pp. 17–50. Available: https://www.aseanlawassociation.org/wp-content/uploads/2019/11/ALA-INDO-legal-system-Part-2.pdf

[22] Raihana S, Alam A, Chad N, Huda TM, Dibley MJ. Delayed Initiation of Breastfeeding and Role of Mode and Place of Childbirth: Evidence from Health Surveys in 58 Low- and Middle- Income Countries (2012–2017). Int J Environ Res Public Health. 2021;18: 5976. doi: 10.3390/ijerph18115976 34199564 PMC8199672

[23] Cohen SS, Alexander DD, Krebs NF, Young BE, Cabana MD, Erdmann P, et al. Factors Associated with Breastfeeding Initiation and Continuation: A Meta-Analysis. J Pediatr. 2018;203: 190–196.e21. doi: 10.1016/j.jpeds.2018.08.008 30293638

[24] Sharma IK, Byrne A. Early initiation of breastfeeding: a systematic literature review of factors and barriers in South Asia. Int Breastfeed J. 2016;11: 17. doi: 10.1186/s13006-016-0076-7 27330542 PMC4912741

[25] Nguyen PH, Frongillo EA, Kim SS, Zongrone AA, Jilani A, Tran LM, et al. Information Diffusion and Social Norms Are Associated with Infant and Young Child Feeding Practices in Bangladesh. J Nutr. 2019;149: 2034–2045. doi: 10.1093/jn/nxz167 31396621 PMC6825823

[26] Statistics Indonesia (Badan Pusat Statistik—BPS), Macro International. Indonesia Demographic and Health Survey 2007. Calverton, Maryland, USA: BPS and Macro International; 2008.

[27] National Population and Family Planning Board (BKKBN), Statistics Indonesia (BPS), Ministry of Health (Kemenkes), ICF. Indonesia Demographic and Health Survey 2012. Jakarta, Indonesia: BKKBN, BPS, Kemenkes, and ICF; 2012.

[28] National Population and Family Planning Board (BKKBN), Statistics Indonesia (BPS), Ministry of Health (Kemenkes), ICF. Indonesia Demographic and Health Survey 2017. Jakarta, Indonesia: BKKBN, BPS, Kemenkes, and ICF; 2018.

[29] World Health Organization, United Nations Children’s Fund (UNICEF). Indicators for assessing infant and young child feeding practices: definitions and measurement methods. Geneva: World Health Organization; 2021. Available: https://apps.who.int/iris/handle/10665/340706

[30] Powers DA, Yoshioka H, Yun M-S. Mvdcmp: Multivariate Decomposition for Nonlinear Response Models. The Stata Journal. 2012;11: 556–576. doi: 10.1177/1536867X1201100404

[31] Hadisuyatmana S, Has EMM, Sebayang SK, Efendi F, Astutik E, Kuswanto H, et al. Women’s Empowerment and Determinants of Early Initiation of Breastfeeding: A Scoping Review. J Pediatr Nurs. 2021;56: e77–e92. doi: 10.1016/j.pedn.2020.08.004 32855004

[32] Nurokhmah S, Rahmawaty S, Puspitasari DI. Determinants of Optimal Breastfeeding Practices in Indonesia: Findings From the 2017 Indonesia Demographic Health Survey. J Prev Med Pub Health. 2022;55: 182–192. doi: 10.3961/jpmph.21.448 35391530 PMC8995937

[33] Harriott RM, Haile ZT, Chertok IRA, Haider MR. Association between place of birth and timely breastfeeding initiation among Cambodian women: a population-based study. Int Breastfeed J. 2022;17: 54. doi: 10.1186/s13006-022-00496-3 35871076 PMC9308348

[34] Global Breastfeeding Collective, UNICEF, WHO. Global breastfeeding scorecard 2022: protecting breastfeeding through further investments and policy actions. 2022. Available: https://www.globalbreastfeedingcollective.org/media/1921/file

[35] Newhook JT, Ludlow V, Newhook LA, Bonia K, Goodridge JM, Twells L. Infant-Feeding among Low-Income Women: The Social Context that Shapes their Perspectives and Experiences. Can J Nurs Res. 2013;45: 28–49. doi: 10.1177/084456211304500303 24236370

[36] Scott JA, Binns CW. Factors associated with the initiation and duration of breastfeeding: a review of the literature. Breastfeed Rev Prof Publ Nurs Mothers Assoc Aust. 1999;7: 5–16. 10197366

[37] Mallick L, Wang W, Farid S, Pullum T. Initiation of Breastfeeding in Low- and Middle-Income Countries: A Time-to-Event Analysis. Glob Health Sci Pract. 2021;9: 308–317. doi: 10.9745/GHSP-D-20-00361 34019481 PMC8324198

[38] Boerma T, Ronsmans C, Melesse DY, Barros AJD, Barros FC, Juan L, et al. Global epidemiology of use of and disparities in caesarean sections. The Lancet. 2018;392: 1341–1348. doi: 10.1016/S0140-6736(18)31928-7 30322584

[39] Birhan TY, Seretew WS, Alene M. Trends and determinants of breastfeeding within one hour in Ethiopia, further analysis of Ethiopian Demographic and Health Survey: multivariate decomposition analysis. Ital J Pediatr. 2021;47: 77. doi: 10.1186/s13052-021-01032-5 33771215 PMC8004466

[40] Vogel JP, Betrán AP, Vindevoghel N, Souza JP, Torloni MR, Zhang J, et al. Use of the Robson classification to assess caesarean section trends in 21 countries: a secondary analysis of two WHO multicountry surveys. Lancet Glob Health. 2015;3: e260–e270. doi: 10.1016/S2214-109X(15)70094-X 25866355

[41] Ahinkorah BO, Aboagye RG, Seidu A-A, Okyere J, Mohammed A, Chattu VK, et al. Rural–urban disparities in caesarean deliveries in sub-Saharan Africa: a multivariate non-linear decomposition modelling of Demographic and Health Survey data. BMC Pregnancy Childbirth. 2022;22: 709. doi: 10.1186/s12884-022-04992-6 36115842 PMC9482294

[42] Opiyo N, Young C, Requejo JH, Erdman J, Bales S, Betrán AP. Reducing unnecessary caesarean sections: scoping review of financial and regulatory interventions. Reprod Health. 2020;17: 133. doi: 10.1186/s12978-020-00983-y 32867791 PMC7457477

[43] Dudeja S, Sikka P, Jain K, Suri V, Kumar P. Improving First-hour Breastfeeding Initiation Rate After Cesarean Deliveries: A Quality Improvement Study. Indian Pediatr. 2018;55: 761–764. doi: 10.1007/s13312-018-1376-3 30345980

[44] Dhami M, Ogbo F, Akombi-Inyang B, Torome R, Agho K, on behalf of the Global Maternal and Child Health Research Collaboration (GloMACH). Understanding the Enablers and Barriers to Appropriate Infants and Young Child Feeding Practices in India: A Systematic Review. Nutrients. 2021;13: 825. doi: 10.3390/nu13030825 33801545 PMC7998710

[45] Hector D, King L, Webb K, Heywood P. Factors affecting breastfeeding practices: applying a conceptual framework. New South Wales Public Health Bull. 2005;16: 52. doi: 10.1071/nb05013 16106273

[46] Kanhadilok S, McGrath JM. An Integrative Review of Factors Influencing Breastfeeding in Adolescent Mothers. J Perinat Educ. 2015;24: 119–127. doi: 10.1891/1946-6560.24.2.119 26957895 PMC4744340

[47] Saniel OP, Pepito VCF, Amit AML. Effectiveness of peer counseling and membership in breastfeeding support groups in promoting optimal breastfeeding behaviors in the Philippines. Int Breastfeed J. 2021;16: 53. doi: 10.1186/s13006-021-00400-5 34247624 PMC8274007

[48] Bongaarts J, Hodgson D. Fertility Trends in the Developing World, 1950–2020. Fertility Transition in the Developing World. Cham: Springer International Publishing; 2022. pp. 1–14. doi: 10.1007/978-3-031-11840-1_1

[49] Benedict RK, Craig HC, Torlesse H, Stoltzfus RJ. Trends and predictors of optimal breastfeeding among children 0–23 months, South Asia: Analysis of national survey data. Matern Child Nutr. 2018;14 Suppl 4: e12698. doi: 10.1111/mcn.12698 30499250 PMC6519202

[50] Yadanar Y, Mya KS, Witvorapong N. Determinants of breastfeeding practices in Myanmar: Results from the latest nationally representative survey. PloS One. 2020;15: e0239515. doi: 10.1371/journal.pone.0239515 32970726 PMC7514058

[51] Flaherman VJ, Chan S, Desai R, Agung FH, Hartati H, Yelda F. Barriers to exclusive breast-feeding in Indonesian hospitals: a qualitative study of early infant feeding practices. Public Health Nutr. 2018;21: 2689–2697. doi: 10.1017/S1368980018001453 29973298 PMC10260846

[52] Choirunisa S, Adisasmita A, Izati YN, Pratomo H, Iriani D. Kangaroo mother care practices for low birthweight newborns in a district hospital in Indonesia. Child Health Nurs Res. 2021;27: 354–364. doi: 10.4094/chnr.2021.27.4.354 35004523 PMC8650954

[53] Titaley CR, Loh PC, Prasetyo S, Ariawan I, Shankar AH. Socio-economic factors and use of maternal health services are associated with delayed initiation and non-exclusive breastfeeding in Indonesia: secondary analysis of Indonesia Demographic and Health Surveys 2002/2003 and 2007. Asia Pac J Clin Nutr. 2014;23: 91–104. doi: 10.6133/apjcn.2014.23.1.18 24561977

[54] Klaten Regency. Regional Regulation (PERDA) concerning Amendment to Klaten Regency Regional Regulation Number 7 Year 2008 on Early Initiation of Breastfeeding and Exclusive Breastfeeding. LD.2019/NO.6 Aug 12, 2019 p. 18. Available: https://peraturan.bpk.go.id/Home/Details/165643/perda-kab-klaten-no-6-tahun-2019

[55] Syam A, Abdul-Mumin KH, Iskandar I. What Mother, Midwives, and Traditional Birth Helper Said About Early Initiation of Breastfeeding in Buginese-Bajo Culture. SAGE Open Nurs. 2021;7: 23779608211040287. doi: 10.1177/23779608211040287 34782864 PMC8590383

[56] Cahyadi N, Hanna R, Olken BA, Prima RA, Satriawan E, Syamsulhakim E. Cumulative Impacts of Conditional Cash Transfer Programs: Experimental Evidence from Indonesia. Am Econ J Econ Policy. 2020;12: 88–110. doi: 10.1257/pol.20190245

[57] Pelto GH. Perspectives on Infant Feeding: Decision-Making and Ecology. Food Nutr Bull. 1981;3: 1–15. doi: 10.1177/156482658100300304

[58] ASEAN, UNICEF, WFP. ASEAN Food and Nutrition Security Report 2021. Jakarta: UNICEF; 2022. Available: https://www.unicef.org/eap/reports/asean-food-and-nutrition-security-report-2021

